# New *In Vitro* Phenotypic Assay for Epilepsy: Fluorescent Measurement of Synchronized Neuronal Calcium Oscillations

**DOI:** 10.1371/journal.pone.0084755

**Published:** 2014-01-08

**Authors:** Nathalie Pacico, Ana Mingorance-Le Meur

**Affiliations:** Neurosciences Therapeutic Area, New Medicines, UCB Pharma, Braine-L’Alleud, Belgium; University of Texas Health Science Center, United States of America

## Abstract

Research in the epilepsy field is moving from a primary focus on controlling seizures to addressing disease pathophysiology. This requires the adoption of resource- and time-consuming animal models of chronic epilepsy which are no longer able to sustain the testing of even moderate numbers of compounds. Therefore, new *in vitro* functional assays of epilepsy are needed that are able to provide a medium throughput while still preserving sufficient biological context to allow for the identification of compounds with new modes of action. Here we describe a robust and simple fluorescence-based calcium assay to measure epileptiform network activity using rat primary cortical cultures in a 96-well format. The assay measures synchronized intracellular calcium oscillations occurring in the population of primary neurons and is amenable to medium throughput screening. We have adapted this assay format to the low magnesium and the 4-aminopyridine epilepsy models and confirmed the contribution of voltage-gated ion channels and AMPA, NMDA and GABA receptors to epileptiform activity in both models. We have also evaluated its translatability using a panel of antiepileptic drugs with a variety of modes of action. Given its throughput and translatability, the calcium oscillations assay bridges the gap between simplified target-based screenings and compound testing in animal models of epilepsy. This phenotypic assay also has the potential to be used directly as a functional screen to help identify novel antiepileptic compounds with new modes of action, as well as pathways with previously unknown contribution to disease pathophysiology.

## Introduction

The enormous complexity of the human nervous system represents a barrier for modern drug discovery. Advances in combinatorial chemistry and high-throughput screening (HTS) technologies mean that high-throughput target-based screening assays are frequently used to identify potential drug candidates [1]. This creates a gap between simplified target-based screenings and *in vivo* animal models of human central nervous system (CNS) diseases. Consequently, candidate compounds have a high dropout rate in this transition. The development of *in vitro* phenotypic assays can help bridge this gap by combining moderate throughput with enough biological context to capture some of the molecular mechanisms of the disease [2]. Given the demands and the difficulty of working with animal models of CNS diseases, *in vitro* phenotypic assays have potential utility as screens for compounds before these are progressed to *in vivo* testing [1].

The field of epilepsy is perhaps one of the best examples of the benefit of using animal models for screening compounds. Although many modern antiepileptic drugs (AEDs) have been discovered using target-based approaches, much of the success of this field comes from the early development of animal seizure models [3]–[5]. However, as the field moves from a primary focus of controlling seizures to the development of drugs able to address aspects of disease pathophysiology [6]–[8], this requires the adoption of much resource- and time-consuming chronic animal models that can longer sustain the testing of even moderate numbers of compounds [9]. *In vitro* models of epilepsy largely rely on electrophysiological measurements of epileptiform activity in brain slices or dissociated neuronal cultures [10], [11]. Such readouts impose significant throughput limitations, making these assays more suitable for mode of action studies than for compound identification or secondary screening. Consequently, there is a need for new *in vitro* functional epilepsy assays able to provide a medium throughput while still preserving sufficient biological context to allow for the identification of compounds with new modes of action.

An alternative approach for measuring neuronal activity is the use of fluorescent probes to monitor the fluctuations of intracellular calcium levels that accompany neuronal depolarization [12]. Image-based measurement of intracellular calcium levels relies on single neuron analysis and has therefore similar throughput limitations as electrophysiological recordings. Fluorescent plate reader-based calcium assays, on the contrary, are amenable to medium or high throughput screening [12]–[14], but can only measure fluctuations of intracellular calcium levels when these occur in a large number of cells. Interestingly, such synchronized intracellular calcium oscillations are reported to occur in high density primary cultures of the hippocampus and cortex, either in a spontaneous fashion or in response to triggering factors such as incubation in low magnesium buffer [15]–[18]. Dual intracellular recordings from multiple neurons and calcium imaging have established that calcium oscillations are strictly associated with bursts of action potentials in these neurons representing epileptiform activity [11], [19], [20]. The same phenomenon has also been reported in hippocampal slices, where it is known that each calcium oscillation corresponds to an individual ictal event experienced by most neurons in the network in a synchronized manner [21]. Despite the high potential of such medium-throughout fluorescent assays, they have only been described in a small number of publications and have not yet been characterized as a predictive tool for AED discovery.

Here we describe a robust and simple fluorescence-based calcium assay to measure epileptiform network activity using rat primary cortical cultures in a 96-well format. We have adapted two well-known cellular models of epileptiform activity to this assay format –low magnesium and 4-aminopyridine models –, and evaluated the contribution of sodium and calcium voltage-gated ion channels and AMPA, NMDA and GABA receptors to epileptiform activity in both models. We have also evaluated their translatability using a panel of AEDs with a variety of modes of action. The *in vitro* phenotypic assay we describe here has a great potential as a functional screen for antiepileptic activity to help identifying compounds with new modes of action and pathways with previously unknown contribution to epilepsy.

## Results

### High density cortical cultures develop spontaneous calcium oscillations

Cortical and hippocampal primary cultures from mouse or rat tissue have been described to develop spontaneous intracellular calcium oscillations when cultured at high density. These oscillations occur throughout the entire population of primary neurons in a pattern that resembles neuronal hyper-synchrony during epileptiform activity [11], [15]–[20]. To determine the extent to which neuronal calcium oscillations can be used as an *in vitro* model of epileptiform activity, we started by reproducing these observations in our laboratory. We loaded primary cultures from rat cortex with the fluorescence calcium indicator Fluo-4 and observed their spontaneous behavior using a fluorescent plate reader (Flexstation). We evaluated cultures at different stages of development (6–20 days *in vitro*) and at different densities by monitoring their activity over 10-minute periods with data acquisition every 0.8 seconds. In early experiments we determined that this short, sub-second, acquisition timing was needed not to miss the peak of the oscillations.

**Figure 1:**
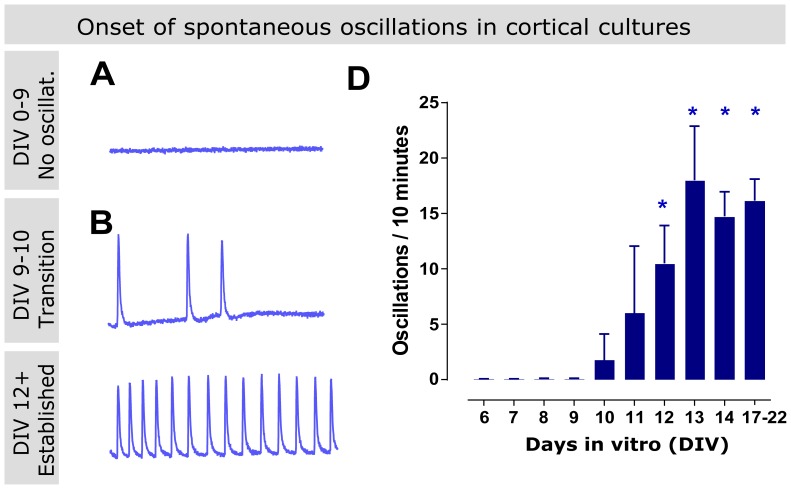
High-density cortical cultures develop spontaneous intracellular calcium oscillations. Primary cultures from rat cortex were loaded with the intracellular calcium reporter dye Fluo-4 and imaged in a fluorescent plate reader. Representative images of 10-minute recordings are used for A-C. (A) Up to 9 days *in vitro* (DIV), the cellular population intracellular calcium level remained constant, with no peak observed during 10-minute recordings. (B) around days 9 and 10, fast elevations in intracellular calcium levels could be detected in some wells. (C-D) After 11-12 DIV the phenotype of calcium oscillations becomes established, with cultures displaying more frequent and rhythmic oscillations. (D) Graph showing the onset of calcium oscillations quantified as number of oscillations per 10-minute recording after following cultures from 3 separate batches of neurons (2 wells per batch and day, minimum 4 wells per time point) over a period of 8 days, from 6 to 14 DIV, and during their third week *in vitro*. Asterisk denotes sample statistically different from zero (one sample t-test, p<0.05).

In high density neuronal cultures (50,000 cells per well in 96-well plates), the spontaneous development of intracellular calcium oscillations occurred around days 9–10 (Figure 1). The pattern of the oscillations among the wells was variable, with some showing no oscillations and others producing a limited number of oscillations during the 10-minute recording (Figure 1B and D). After 13 days *in vitro* the calcium oscillations pattern reached a plateau and stabilized at around 15 oscillations per 10-minute recording (Figure 1C and D). Cultures younger than 9 days failed to show any calcium oscillation (Figure 1A and D).

While the number of oscillations per well was variable, their frequency was constant for any given well, allowing for pharmacological studies aimed at modulating this frequency.

Cultures plated at a slightly lower density (40,000 cells per well) had a more variable onset of oscillations but reached a similar plateau. Cultures of 20,000 cells per well or lower failed to produce any oscillations during a period of 20 days or produced a few oscillations in random wells (not shown). We therefore decided to work with cultures at 13–15 days *in vitro* and 50,000 cells per well for all subsequent pharmacological studies.

### Calcium oscillations are affected by compounds that modulate the activity of voltage- and ligand-gated ion channels

In order to determine to how closely synchronized calcium oscillations mimic epileptiform activity, we explored their modulation by low magnesium and 4-aminopyridine (4-AP), two established methods for inducing epileptiform activity in tissue slices [10], [22], [23].

**Figure 2:**
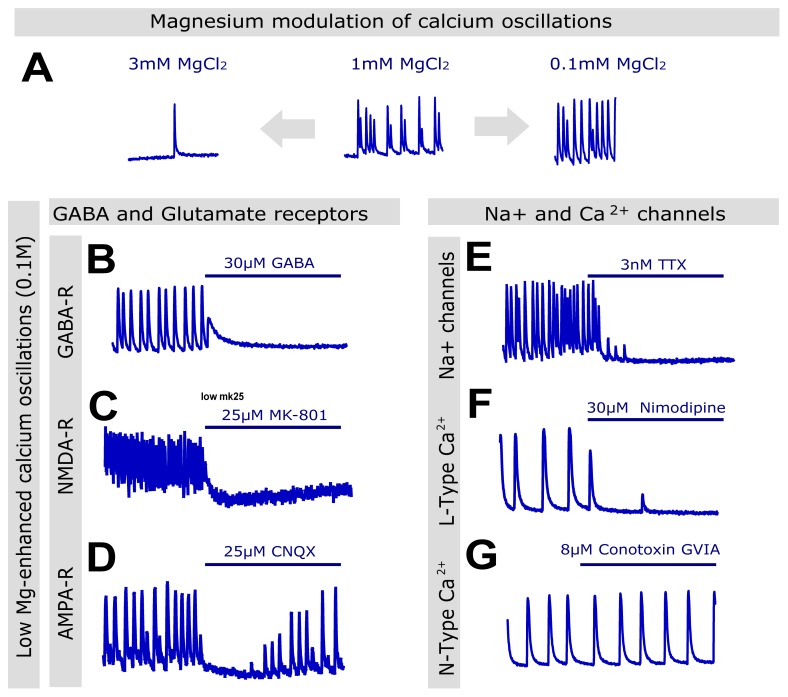
Low magnesium-enhanced calcium oscillations are modulated by GABA and glutamate receptors, and ion channels. (A) Representative recordings of cultures imaged in the presence of normal magnesium levels in the buffer (1 mM, center), high magnesium buffer (3 mM) or low magnesium buffer (0.1 mM). While high magnesium in the buffer arrested or severely reduced the number of oscillations, incubation in 0.1 mM magnesium led to more numerous and stable (similar amplitude) oscillations. (B-D) Response of the low magnesium-enhanced calcium oscillations to GABA receptor agonism (B), NMDA receptor inhibition (MK-501, C) and AMPA receptor inhibition (CNQX, D) injected 4 minutes into the recording (time exposed to the compound denoted by blue bar). (E-G) Response of the calcium oscillations to ion channel inhibitors.

We first evaluated the impact of varying concentrations of magnesium in the recording buffer on spontaneous calcium oscillations (Figure 2A). When high density neuronal cultures were exposed to low magnesium buffer (0.1 mM MgCl_2_), their spontaneous calcium oscillations increased in frequency and became more stable, with most oscillations reaching the same peak fluorescence level. Conversely, when the cultures where exposed to high magnesium concentrations, cultures displayed very few or no oscillations during the 10-minute recording (Figure 2A). These observations are consistent with the ability of extracellular magnesium levels to reduce calcium influx by blocking the NMDA receptor channel [10].

We next evaluated the sensitivity of the calcium oscillations to modulation of the four main molecular targets of AEDs, namely GABA receptors, glutamate receptors, and voltage-gated sodium and calcium channels [24], [25]. For these experiments we recorded the oscillation pattern in each well for a baseline period of 4 minutes, followed by a further 6 minutes after injecting the different compounds into the wells. We also used low magnesium buffer to stabilize the variability in the pattern and frequency of the spontaneous oscillations. Increasing GABAergic tone using 30 µM GABA (Figure 2B) and reducing glutamatergic neurotransmission using selective NMDA and AMPA inhibitors (MK-801 and CNQX at 25µM, respectively; Figure 2C-D) resulted in a decrease or suppression in the appearance of calcium oscillations. Similarly, inhibition of sodium and L-type calcium channels using 3 nM TTX and 30 µM nimodipine, respectively, suppressed the calcium oscillations (Figure 2E-F). Inhibition of N-type calcium channels by omega-conotoxin GVIA, even at high concentrations (8µM) had little impact on the oscillations, Figure 2G). These findings are consistent with the ability of magnesium to modulate the oscillations frequency as reported previously [15].

**Figure 3:**
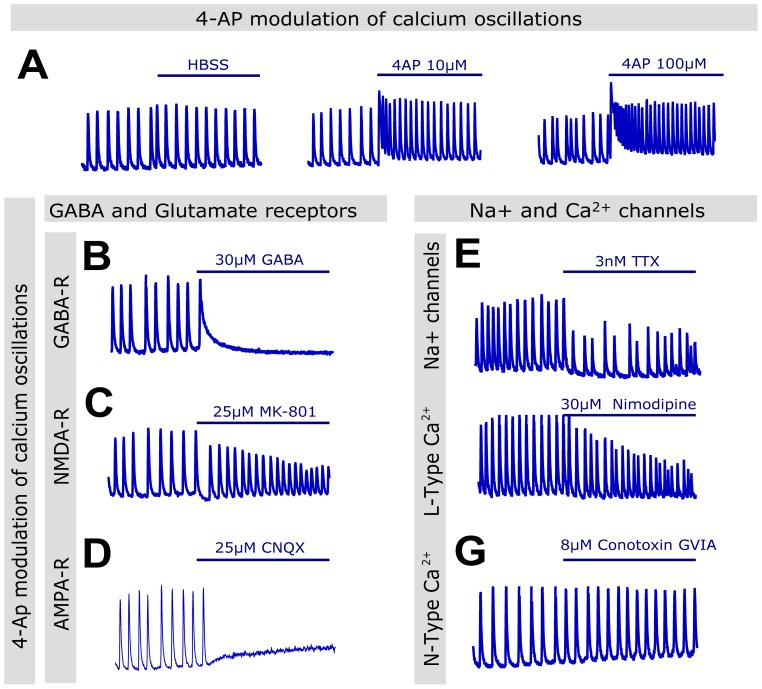
4-AP-enhanced calcium oscillations are modulated by GABA and glutamate receptors, and ion channels. (A) Representative recordings of cultures exposed to vehicle or 4-AP 4 minutes into the recording (time exposed to the compound denoted by blue bar is 6 minutes). 4-AP induced a dose-response transient elevation of intracellular calcium baseline accompanied by an increase in frequency. (B-D) Response of the 4-AP-enhanced calcium oscillations to GABA receptor agonism (B), NMDA receptor inhibition (MK-501, C) and AMPA receptor inhibition (CNQX, D). (E-G) Response of the calcium oscillations to ion channel inhibitors.

We also explored the modulation of calcium oscillations by 4-AP, widely reported in the literature to trigger epileptiform activity in non-epileptic tissues ([10], [22], [23] Figure 3). In our assay, application of 4-AP resulted in an increase in basal intracellular calcium levels in a dose-response, but transient manner. As the calcium levels gradually returned to baseline, we observed a concomitant increase in the frequency of calcium oscillations that remained for the duration of the 10-minute recording (Figure 3A).

Similarly to the low magnesium model, 4-AP-enhanced calcium oscillations were sensitive to the same compounds that reduced or inhibited calcium oscillations in the low magnesium model, namely GABA, MK-801, CNQX, TTX and nimodipine, while they were insensitive to omega-conotoxin GVIA (Figure 3B-G). 4-AP-enhanced calcium oscillations had a tendency to be more resistant than low magnesium-enhanced calcium oscillations, with doses of compounds that completely suppressed low magnesium-enhanced calcium oscillations producing only partial reduction in the model. Nevertheless, all the modes of action tested suppressed both low magnesium- and 4-AP-enhanced calcium oscillations at higher compound concentration with the exception of omega-conotoxin GVIA (Figure 2-3). Collectively, these data confirm the involvement of voltage-and ligand-gated ion channels known to control neuronal excitability in the generation of synchronized calcium oscillations *in vitro* and suggest this assay could mimic the mechanisms involved in generating epileptiform activity.

### Neuronal calcium oscillations respond to a variety of antiepileptic drugs

**Figure 4:**
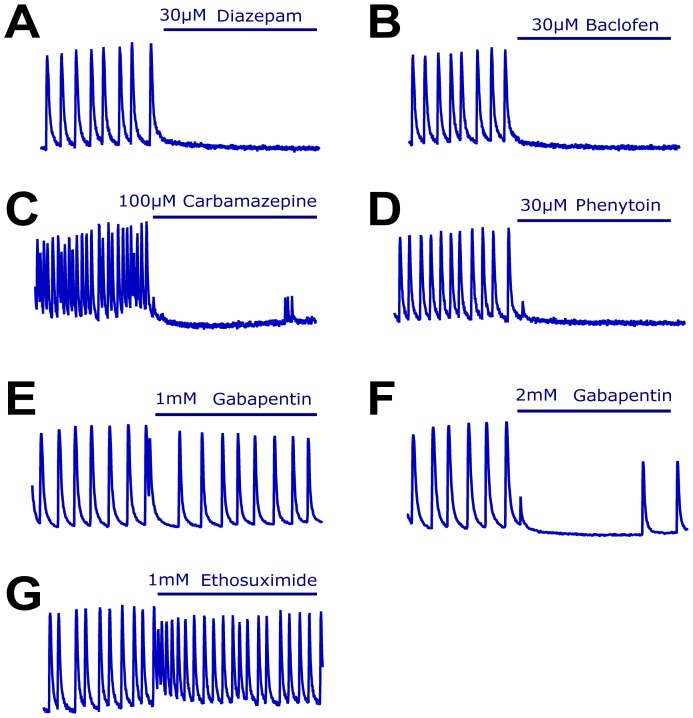
Changes in Calcium oscillations in response to application of classic antiepileptic drugs. (A-G) Representative recordings of cultures exposed to antiepileptic drugs that work by enhancing GABA receptor activity (A-B), inhibiting sodium channels (C-D) and inhibiting calcium channels (E-G). While other AEDs showed activity in the model, ethosuximide turned from inactive to excitatory at doses higher than 500µM. Time exposed to the compound denoted by blue bar is 6 minutes.

**Figure 5:**
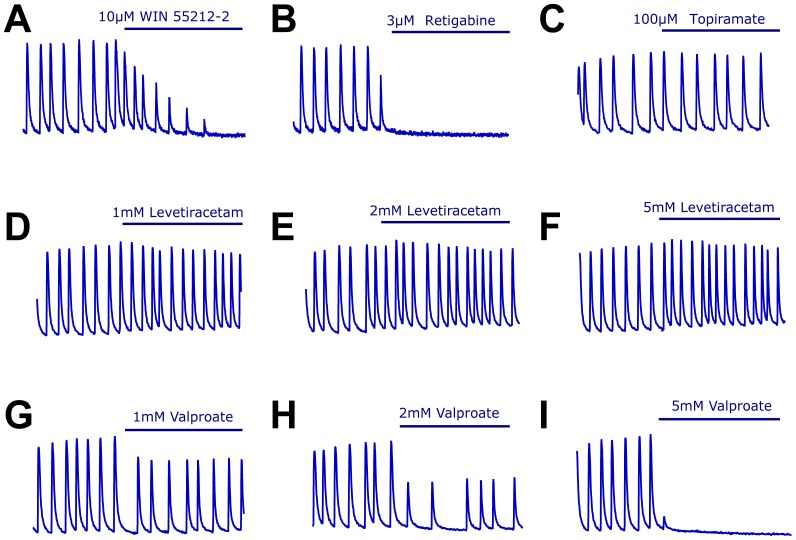
Changes in calcium oscillations in response to application of antiepileptic drugs working through a variety of modes of action. (A-E) Representative recordings of cultures exposed to an agonist of cannabinoid CB1 receptors (A), a potassium channel opener (B), showing activity in this model Topiramate was inactive at the highest dose that could be tested (C). Levetiracetam also failed to show activity in this model at concentrations up to 5 mM (D-E) while valproate showed partial activity at concentrations up to 2 mM (G-H) and suppression of calcium oscillations at 5 mM (I). Time exposed to the compound denoted by blue bar is 6 minutes.

In order to be used as a secondary screen and predictive tool for the discovery of antiepileptic compounds, the epileptiform-like phenotype displayed by the high density cortical cultures must be relevant to the human disease. To better understand the translatability of this *in vitro* phenotypic assay, we evaluated the ability of a panel of approved AEDs with a variety of modes of action to reduce the expression of calcium oscillations (Figures 4 and 5). Because low magnesium-induced calcium oscillations were more sensitive to compounds targeting ion channels and receptors than those enhanced by 4-AP, we profiled the panel of AEDs in this model.

We first evaluated AEDs known to act through classic modes of action, namely by activating GABA receptors or inhibiting sodium and calcium voltage-gated channels (Figure 4) [25]. Administration of GABA_A_ and GABA_B_ receptor agonists (30µM diazepam and 30µM baclofen, respectively) suppressed the formation of calcium oscillations (Figure 4A–B). The sodium channel inhibitors carbamazepine and phenytoin were also able to suppress the formation of calcium oscillations (100 and 30µM respectively; Figure 4C-D). In contrast, gabapentin was only able to reduce the generation of calcium oscillations when used at high concentrations (2 mM, Figure E-F). In the case of a second AED also thought to have efficacy by modulating calcium channels, ethosuximide, there was a trend towards exacerbating the calcium oscillations when applied at high doses, in a similar manner to 4-AP (Figure 4G) and no activity at lower doses. These observations suggest some of the main AED targets, but not all, are able to modulate calcium oscillations in this assay.

A number of AEDs or compounds with anticonvulsant properties in rodents are believed to act through other, non-classic, modes of action [24]. Application of the cannabinoid receptor agonist WIN 55212-2 resulted in a gradually decrease on calcium oscillations at 10µM, the highest dose that could be tested due to solubility limitations (Figure 5A) suggesting CB1 receptors are expressed in the mixed cortical cultures and contribute to regulating neuronal excitability. The potassium channel opener retigabine also had efficacy in the oscillation assay at low µM concentrations(3 µM; Figure 5B). In contrast, the calcium oscillation assay was not sensitive to other approved AEDs such as topiramate and levetiracetam at the highest dose that could be tested for each compound (Figure 5C and D-F), and in the case of valproate, it only showed activity at very high concentrations (mM range, Figure 5G-I). These results are summarized in Table 1. Overall these data suggest the fluorescence-based calcium oscillations assay captures several signaling pathways that are involved in controlling neuronal excitability including a number of targets for known antiepileptic drugs.

**Table 1:** Summary of the compounds and antiepileptic drugs evaluated in the calcium oscillations assay.

Compound	Mechanism	Modulation of calcium oscillations
GABA	GABA receptor agonist	+
Diazepam	GABA_A_ receptor agonist	+
Baclofen	GABA_B_ receptor agonist	+
TTX	Na^+^ channel inhibitor	+
Carbamazepine	Na^+^ channel inhibitor	+
Phenytoin	Na^+^ channel inhibitor	+
Nimodipine	L-type Ca^+2^ channel inhibitor	+
Conotoxin GVIA	N-type Ca^+2^ channel inhibitor	−
Ethosuximide	T-type Ca^+2^ channel inhibitor	−
Gabapentin	Alpha2delta-1 Ca^+2^ channel inhibitor	+
MK-801	NMDA receptor inhibitor	+
CNQX	AMPA/KA receptor inhibitor	+
WIN 55212-2	Cannabinoid receptor agonist	+
Retigabine	K^+^ channel opener	+
Levetiracetam	SV2A ligand	−
Topiramate	Multiple mechanisms	−
Valproate	Multiple mechanisms	+

## Discussion

As the epilepsy field moves from a primary focus on controlling seizures to addressing disease pathophysiology there is a need for new *in vitro* functional assays able to balance throughput and translatability. Here we describe a phenotypic cell-based assay using rat cortical cultures that captures several of the mechanisms involved in epileptiform activity, including those through which numerous approved AEDs exert their therapeutic activity. Because of its throughput and translatability, the calcium oscillations assay has the potential to be used for screening compounds with potential antiepileptic activity before *in vivo* testing.

Studies reported in the literature have shown that hippocampal and cortical cultures develop spontaneous intracellular calcium oscillations that resemble epileptiform activity [15], [16], [18], [19], [26]. A well-defined phenotype is the single most important consideration when designing a phenotypic assay or screen [2]. Our data confirm and extend previous observations that glutamate and GABA receptors are involved in the formation of the spontaneous calcium oscillations [15], [17]–[20]. We observed enhanced sensitivity to NMDA receptor blockade in the low magnesium model compared with the 4-AP model, with doses of MK-801 able to suppress calcium oscillations in the former providing only partial suppression in the latter. Interestingly we observed the reverse pattern in the case of the AMPA and kainate receptor inhibitor CNQX, which had preferential activity in the 4-AP model. These observations are consistent with previous descriptions of the neuronal oscillations model using calcium imaging by Wang and Gruenstein [17]. In their study, Wang and Gruenstein also observed the necessity of L-type, but not N-type, calcium channels for the continuation of calcium oscillations in cortical cultures that we observed in our plate reader assay [17]. Also supporting our observations, synchronized calcium oscillations are described to be sensitive to the sodium channel blocker TTX and to the L-type calcium channel blocker nimodipine [15], [19], [20]. Therefore, we confirm in our study that a majority of targets associated with the mode of action of classic AEDs appear to be involved in the generation of calcium oscillations in cortical cultures.

To evaluate the translatability of this assay, we tested the ability of a panel of AEDs with a diverse set of modes of actions to suppress the formation of calcium oscillations. While there is *in vitro* data for all of these drugs, many have been profiled in rat hippocampal slices by electrophysiology and not in primary culture models of epileptiform activity. Compounds that were active in our assay have also been reported to have activity either in similar primary culture assays (WIN 55212-2, baclofen [11], [18], [27]) or in the low magnesium model in slices (carbamazepine, phenytoin and retigabine [28]–[30]). Interestingly ethosuximide, which failed to inhibit calcium oscillations in our assay, was also inactive up to 1 mM in a similar assay using hippocampal neurons [11], confirming our observations.

Some AEDs with proven efficacy for certain types of epilepsy were unable to suppress the phenomenon of calcium oscillations in this assay. In the case of the synaptic vesicle 2A protein ligand levetiracetam, previous studies using tissue slices have shown that its activity might depend on the specific model tested, being reported to reduce epileptiform activity in human neocortical slices exposed to the GABA receptor inhibitor bicuculline but not when epileptiform activity was induced by low magnesium [31]. While the activity of levetiracetam in the first model was reported at 100–500µM, concentrations up to 1 mM were inactive in the low magnesium slice model [31], consistent with our observations using primary cortical neurons with concentrations up to 5 mM. Topiramate has also been shown to be active in hippocampal primary neurons where epileptiform burst firing events are induced following transient exposure to a medium with no magnesium at doses that were inactive in our assay [33]. However a potential activity of topiramate in this model cannot be ruled out because the compound solubility limited our ability to evaluate concentrations higher than 100µM. Indeed two other AEDs that were inactive in this assay at micromolar concentrations were able to suppress the calcium oscillations when tested at concentrations above 1 mM. This was the case of gabapentin, which has been shown to be also inactive in the low magnesium model using human neocortical slices at micromolar concentration [32], but to be partly active in a combined low magnesium high potassium model using rat hippocampal slices [28], suggesting its activity might also depend on the specific model being tested. Valproate has been described to have modest activity at 1 mM concentration in the low magnesium model using rat hippocampal slices [28], [29]. In our assay, valproate only produced a small reduction in the calcium oscillations peak amplitude at 1 mM but progressed to complete efficacy when tested at 5 mM. Because slice studies measure epileptiform activity by electrophysiology it is possible that such a read out identifies the anti-epileptiform activity of some compounds before it can be measured in the fluorescent assay. Alternatively, the efficacy of gabapentin and valproate in this assay at high, potentially non-physiological, concentrations might be mediated by receptors other than those driving their antiepileptic activity *in vivo* and in more comprehensive *in vitro* systems such as slice assays. Overall, our head to head comparison of a panel of AEDs indicates the fluorescence-based calcium oscillations assay captures several signaling pathways that are involved in controlling neuronal excitability including a number of targets for known antiepileptic drugs and is comparable to other *in vitro* epilepsy assays.

For those pathways that are conserved in the mixed cortical cultures, this assay has significant potential as a predictive tool for AED discovery and evaluation. Not only does it offers a fast response and medium throughput, but it can also be used to obtain early *in vitro* proof of concept with compounds that are not suitable for *in vivo* testing due to poor pharmacokinetic properties or via genetic modulation such as target knockdown [34]. The nature of the mixed cortical cultures also offers the possibility of evaluating the role of glial proteins in the generation of epileptiform activity [35]. For all of these pathways, *in vitro* phenotypic assays can help bridge the gap between primary target-based screening and *in vivo* testing and be used to characterize and preselect the compounds that will progress through the drug discovery cascade.

Nevertheless, the calcium oscillations assay shows some degree of drug refractoriness, in particular to those AEDs that are believed to have complex modes of action ([24], Table 1). A number of reasons might explain this observation. First, some of the targets of these AEDs might not be expressed in the cultures or not contribute substantially to the epileptiform activity. This might be the case of the N-type calcium channels. Second, some important ligands or factors that are needed to engage those targets might be missing in these cultures, thereby making modulators of those targets ineffective in the assay. Last, some important aspects of epilepsy are not cell autonomous and might require an appropriate circuit connectivity that is lacking in the dissociated cultures. For these modes of action, the calcium oscillations model might not sufficiently capture the complexity of the disease and highlights the need to use different models when evaluating the potential of a candidate drug [36].

Some degree of drug refractoriness is also observed in animal models of epilepsy, where some models or subgroups of animals show a poor response to AEDs [37]. The specific molecular and cellular mechanisms that lead to pharmacoresistant forms of epilepsy remain largely unknown [6], [7]. This lack of knowledge represents a barrier for the development of target-based drug screening for refractory epilepsy. The phenotypic calcium oscillations assay that we describe here offers an interesting opportunity by allowing the primary screening of compounds with potential efficacy in pharmacoresistant epilepsy without previous information about their biochemical targets. Whether phenotypic screens using this assay can successfully identify compounds with efficacy in rodent models and patients with pharmacoresistant epilepsy remains to be determined.

In addition to being used as primary and secondary screens, phenotypic assays can be used to better understand the mechanism behind their disease-relevant phenotype and shed light on the pathology that they model. In the case of the calcium oscillations assay, future research on the process leading to the onset of the oscillations *in vitro* might also provide useful information for understanding the mechanisms underlying epileptogenesis. Overall, the fluorescent-based phenotypic assay that we have characterized has the potential to be used as a predictive tool for AED discovery and to bridge the gap between target-based screening and *in vivo* testing. Given the demands and difficulties of working with animal models of CNS diseases, such *in vitro* phenotypic assay can bring great value to the discovery of AEDs.

## Materials and Methods

### Ethics statement

All experiments involving animals were approved by the UCB ethical committee for animal experimentation, in accordance with the European Directive 2010/63/EU on the protection of animals used for scientific purpose and with the Belgian law on the use of laboratory animals.

### Reagents

Fluo-4 non wash was purchased from Invitrogen. Hank's balanced salt solution (HBSS), Hepes, CaCl_2_ and MgCl_2_ from Lonza. Diazepam was from Fragon; phenytoin from Fluka; conotoxin GVIA and TTX from Alomone Labs; gabapentin from TCI Chemicals; and levetiracetam and retigabine were synthetized at UCB. All other reagents were from Sigma Aldrich.

### Primary neurons

Cortical cultures were prepared from rat embryos (OFA) at 17–18 days postcoitum and were cultured on poly-L-lysine coated 96-well plates (Greiner) following a protocol adapted from Mingorance-Le Meur and O’Connor (2009). Brain cortex was collected in ice-cold PBS and dissociated in 0.25% trypsin (Invitrogen) in PBS for 20 minutes in the incubator. Trypsin activity was then stopped by adding serum-containing plating medium consisting of neurobasal, 1% penicillin-streptomycin, 2% B27, 0.25% glutamax and 10% normal horse serum (Invitrogen). After inactivation the tissue was collected with a centrifugation step, resuspended in 1 ml of plating medium and viable cells were counted using a cell counter before being plated at 50,000 cells/well in plating medium. Four hours after plating, the medium was changed to serum free maintenance medium based on neurobasal-B27. Three days after plating, half of the medium was renewed and the cultures were maintained with two full media changes per week afterwards. For all pharmacological studies, cultures were used after 14 days *in vitro* DIV.

### Calcium imaging

Incubation with Fluo-4 was performed according to recommendations from the manufacturer (Invitrogen). Briefly, a vial of Fluo-4 was resuspended in HBSS-Hepes 20 mM containing 3 mg/ml BSA and 100µl probenecid and the cultures were incubated in 80µl of Fluo-4 per well for 1 hour at 37°C. Plates were then taken out of the incubator and allowed to reach room temperature before being imaged. For imaging, the Fluo-4-containing HBSS was replaced by 180µl of either normal magnesium HBSS, or calcium and magnesium-free HBSS supplemented with 0.1 mM MgCl_2_ and 2 mM CaCl_2_. Cultures were imaged in this medium.

For calcium imaging, Flexstation 2 and 3 plate readers running SoftMax pro at set at 24°C were used. Fluo-4 fluorescent was imaged with an excitation wavelength of 485 nm, emission of 525 and a cut off at 515. Recording time was 600 seconds with intervals of 0.8 seconds, photomultipliers set at high and 6 reads per well. This allowed for recording of 4 wells (in one column) at a time. For compound injection, a compound transfer protocol was used with a 20µl volume of compound added to the wells at 240 seconds. Compounds were first dissolved in HBSS or DMSO and then diluted to 10-fold final concentration in HBSS for injection. Matching vehicles (HBSS or 0.1 to 0.3% final DMSO in HBSS) were used to monitor a potential injection effect in the calcium oscillations read out. The final compound dose was chosen based on the literature and for those compounds with no activity on the calcium oscillations, higher doses were also tested.

### Data analysis

Fluorescence intensity measurements every 0.8 seconds were transferred to GraphPad Prism for presentation. Effectiveness of the test compounds was assessed in a qualitative way by determining efficacy, partial efficacy or lack of efficacy based on changes after compound addition compared with the baseline before injection. For the first figure showing the onset of calcium oscillations, the number of oscillations per 10 minutes recording was manually counted, then analyzed in GraphPad Prism using a one-sample t-test to determine when they become different from 0.
